# The at-wavelength metrology facility for UV- and XUV-reflection and diffraction optics at BESSY-II

**DOI:** 10.1107/S1600577515020615

**Published:** 2016-01-01

**Authors:** F. Schäfers, P. Bischoff, F. Eggenstein, A. Erko, A. Gaupp, S. Künstner, M. Mast, J.-S. Schmidt, F. Senf, F. Siewert, A. Sokolov, Th. Zeschke

**Affiliations:** a Institute for Nanometre Optics, Helmholtz-Zentrum Berlin, Albert-Einstein-Strasse 15, Berlin 12489, Germany

**Keywords:** reflectometer, c-PGM beamline, at-wavelength metrology, polarimetry, reflectivity, diffraction gratings, XUV optical elements

## Abstract

A technology center for the production of high-precision reflection gratings has been established. Within this project a new optics beamline and a versatile reflectometer for at-wavelength characterization of UV- and XUV-reflection gratings and other (nano-) optical elements has been set up at BESSY-II. The Plane Grating Monochromator beamline operated in collimated light (c-PGM) is equipped with an SX700 monochromator, of which the blazed gratings (600 and 1200 lines mm^−1^) have been recently exchanged for new ones of improved performance produced in-house. Over the operating range from 10 to 2000 eV this beamline has very high spectral purity achieved by (i) a four-mirror arrangement of different coatings which can be inserted into the beam at different angles and (ii) by absorber filters for high-order suppression. Stray light and scattered radiation is removed efficiently by double sets of *in situ* exchangeable apertures and slits. By use of in- and off-plane bending-magnet radiation the beamline can be adjusted to either linear or elliptical polarization. One of the main features of a novel 11-axes reflectometer is the possibility to incorporate real life-sized gratings. The samples are adjustable within six degrees of freedom by a newly developed UHV-tripod system carrying a load up to 4 kg, and the reflectivity can be measured between 0 and 90° incidence angle for both *s*- and *p*-polarization geometry. This novel powerful metrology facility has gone into operation recently and is now open for external users. First results on optical performance and measurements on multilayer gratings will be presented here.

## Abstract 2

A new optics beamline and a versatile 11-axes UHV-reflectometer for at-wavelength characterization of real life-sized UV- and XUV-reflection gratings and other (nano-) optical elements has been set up and is in operation at BESSY-II. Azimuthal rotation of samples allows for reflectometry and polarimetry measurements in *s*- and *p*-polarization.

## 1. Introduction   

At-wavelength metrology is a powerful and indispensable non-destructive tool for the development, characterization and final control of XUV-optical elements (Underwood & Gullikson, 1998 ▸; Gullikson *et al.*, 2001 ▸; Tümmler *et al.*, 2003 ▸; Laubis *et al.*, 2009 ▸). Since the optical constants of the coating materials involved depend on wavelength, information on reflectivity or diffraction efficiency at a certain wavelength can be obtained only by this method and cannot be deduced from any other diagnostics results. Thus this method is complementary to *ex situ* profilometry methods delivering information about figure and finish (slope and roughness) of optical elements (Siewert *et al.*, 2011 ▸, 2013 ▸) or Cu *K*α diffractometry which in the case of multilayers delivers information on interfacial roughness and layer quality.

The Helmholtz-Zentrum Berlin operates a grating technology project for the fabrication of high-precision laminar and blazed gratings (Loechel *et al.*, 2013 ▸; Siewert *et al.*, 2016 ▸). Within this project we have designed and fabricated a versatile UHV-reflectometer for the at-wavelength characterization of the in-house produced gratings, *i.e.* the determination of the diffraction efficiency in the wavelength range of interest.

At-wavelength metrology is the final test drive for optical elements before delivery. Apart from at-wavelength metrology for X-ray optical elements, reflectometry is also a very powerful scientific technique. It allows nondestructive characterization and depth-profiling of microstructures, layered systems and buried interlayers to be carried out (Filatova *et al.*, 2009*a* ▸,*b* ▸). It enables atomic concentration profiles (Filatova *et al.*, 2012 ▸, 2014 ▸) to be reconstructed, polarization dependence and anisotropy effects of helical substances to be investigated (Filatova & Lukyanov, 2002 ▸; Filatova *et al.*, 2005 ▸), and information about roughness of surface and buried interface structures to be obtained (Konyushenko *et al.*, 2014 ▸). Based on accurate reflection coefficient spectra it is possible to calculate the spectral dependence of optical constants (Filatova *et al.*, 1999 ▸, 2009*b* ▸).

Currently at BESSY-II two experimental stations, a small reflectometer (Schäfers & Cimino, 2013 ▸) and a polarimeter (Schäfers *et al.*, 1999 ▸), are in operation, which have been used for many years for at-wavelength reflectometry (Schäfers *et al.*, 1998 ▸; Chkhalo *et al.*, 2013 ▸), polarimetry (MacDonald *et al.*, 2009 ▸; Gaupp *et al.*, 2013 ▸) and ellipsometry (Uschakow *et al.*, 2013 ▸) measurements.

The main feature of the new reflectometer to be presented here is the possibility to incorporate *real* life-sized XUV-gratings into the UHV-chamber. The samples are adjustable within six degrees of freedom by a newly designed compact tripod system and the reflectivity can be measured at any incidence angle for both *s*- and *p*-polarization geometry, which requires an azimuthal rotation of the sample around the beam direction. The reflectometer is located in a moderate air-conditioned clean-room hutch at the experimental floor of BESSY-II and is permanently attached to the Optics Beamline PM-1 which has also been set up recently using an SX700 plane-grating monochromator (PGM) operated in collimated light from a bending magnet (Petersen *et al.*, 1995 ▸; Follath *et al.*, 1998 ▸).

The grating project enabled us to set up a new optics beamline and a new reflectometer as a permanent end-station, which are primarily dedicated to optical metrology, reflectometry and ellipsometry at short-term request. It is also open to external users on the basis of cooperation projects or *via* evaluated scientific beam time proposals. The optical concept and design features of both beamline (Sokolov *et al.*, 2014 ▸) and reflectometer have been discussed previously (Eggenstein *et al.*, 2013 ▸, 2014 ▸).

In this paper we discuss briefly the technical/optical design and the properties of both optics beamline and the reflectometer end-station. Performance data and first experimental results on multilayer gratings and on reflection zone plates are given.

## 2. Optics beamline   

The optics beamline has been set up recently at the BESSY-II dipole DIP 1.1. The design incorporates the experience with approximately 25 c-PGM beamlines operating successfully at BESSY-II. It addresses all requirements for quantitative reflectometry as known from other laboratories:

(i) Large energy range (10–2000 eV) covered with only two gratings.

(ii) Moderate energy resolution (*E*/Δ*E*: 1000–5000).

(iii) Flexible operation of PGM in high-resolution, high-flux or high-order suppression modes.

(iv) Highest spectral purity from higher diffraction orders.

(v) Low divergence of incident beam.

(vi) Moderate focus size.

(vii) Aperture system for suppression of scattered and stray light.

(viii) Polarization steering between linear (in-plane) and elliptical (off-plane).

**Figure 1:**
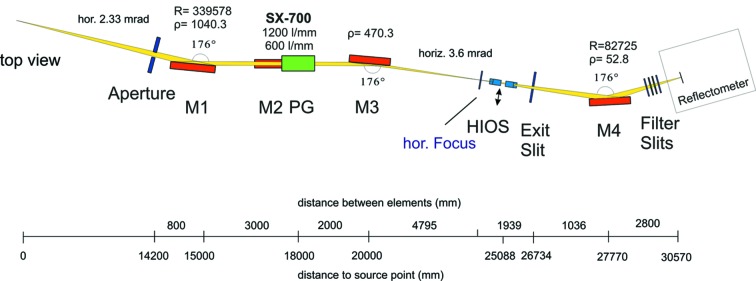
Optical layout of the optics beamline as seen from the top.

The source parameters are listed in Table 1 ▸. Fig. 1 ▸ presents the beamline optical layout. It is equipped with a PGM with a variable plane mirror operated in collimated beam. The beamline acceptance determined by the dimension and incidence angle of the first mirror is 0.5 mrad × 2.33 mrad (v × h). Mirror M1 focuses the incident beam horizontally 10 m downstream in a 1.5:1 demagnification and collimates the beam vertically. For the monochromator, an existing SX700 (PM-1 of BESSY-I) was refurbished (Riemer & Torge, 1983 ▸; Petersen, 1986 ▸) and is equipped with two plane gratings and with a rotatable plane mirror M2. The monochromator operation in collimated light enables a very flexible operation at different *c*_ff_ values or on-blaze (*c*_ff_: fix focus constant), which is the ratio between the cosines of the diffraction angle β and the incident angle α on the grating for the particular diffraction order *m* (*c*_ff_ > 1: inside order, *m* > 0, α > β; *c*_ff_ < 1: outside order, *m* < 0, β > α). Use of blazed gratings enables the operation ‘on-blaze’, at which the desired diffraction order (typically first order) is diffracted specularly with respect to the blaze facets with correspondingly highest efficiency. The dispersed beam is vertically focused by the cylindrical mirror M3 onto the exit slit. The refocusing toroidal mirror M4 focuses the light onto the sample position. As a final conditioning step the beam passes through a filter and slit unit (FSU), a double set of slits and absorption filters and an intensity monitoring system in front of the reflectometer experimental station. All focusing optical elements of the beamline are listed in Table 2 ▸ and the monochromator optics listed in Table 3 ▸.

During the design process the beamline was intensively simulated with the ray-tracing program *RAY* (Schäfers, 2008 ▸). After delivery of the mirrors the simulations were repeated with their real parameters (radii *R* and ρ) and measured slope and finish errors, and the beamline layout (the distances between the optical elements) was adjusted adequately.

The beamline can also be operated with off-plane radiation from the BESSY-II bending magnet. Thus the polarization can be changed from horizontal linear polarization (S1 = +1) to elliptical polarization with a selectable degree of circular polarization (S3 ≤ ±0.8). This is done by an azimuthal rotation of the pre-mirror M1 accompanied by an equal but opposite correction of incidence angle of the monochromator mirror M2 (Kachel *et al.*, 2015 ▸). Thus the incidence angle on the grating is unaffected by this operation and no energy shift is encountered during change of polarization.

For effective high-order suppression, which is essential for quantitative reflectometry, the optics beamline is equipped with two systems which can be inserted into the beam path. The first one is a set of 12 absorber filters installed in a specially designed filter-slit unit chamber (FSU) between refocusing mirror and reflectometer station. Exploiting the transmission cut-off at absorption edges, photons with twice the energy of the first order may be suppressed by a factor of 10–1000. To reach a balance between transmission (>30%) and suppression efficiency most filters have a thickness of 750 nm, except for Al (500 nm) and C_6_H_8_ (1500 nm). The filters are mounted on two feedthroughs. Thus two filters can be inserted into the beam simultaneously, which can be either identical to double the suppression effect or of material combinations to achieve even third- or higher-order suppression.

In the same FSU chamber two sets of interchangeable apertures can be inserted into the beam, one of them upstream and the other downstream of the filters. The size of these apertures allows scattered incident light to be cut off as well as shaping the beam in one (horizontally by slits) or two directions (pinholes). The downstream aperture unit allows for suppression of scattering of the filters.

As an additional option the filters are electrically isolated. Thus measurement of the drain current is foreseen for use of the filters as an *in situ* intensity monitor (Io).

The second high-order suppression system (HiOS) is a four-mirror system (Waki *et al.*, 1989 ▸; Bulicke *et al.*, 1997 ▸; Frommherz *et al.*, 2010 ▸) which can also be inserted into the beam. The first two and the last two mirrors, aligned pairwise parallel to each other on a common platform, are rotated by two goniometers in opposite directions to have no net vertical offset of the reflected beam. The rotation axes of the goniometers lie in the center of the first and last mirror. Therefore the second and third mirrors are longer and the beam glides along the surface while changing the incidence angle. Two sets of four-mirror systems are mounted next to each other on the same translation stage with different vertical offset to realise different angular scan ranges. The high-energy cut-off is freely selectable by the incidence angle chosen, due to drop of reflectivity above the critical angle. This effect is strongly dependent on the optical constants and thus on the coating material. In this way most of the energy range between 20 eV and 700 eV can be covered with a suppression efficiency of 10^4^ while keeping transmission at approximately 40%. Thus the two high-order suppression systems on the optics beamline provide a wide flexibility for light shaping upstream of the reflectometer, of which a properly aligned high-order suppression system is by far more effective than a filter unit.

## 3. 11-axes reflectometer   

The reflectometer has been specified to complement the features of the existing reflectometer and polarimeter with measurement flexibility on realistic large-scale samples together with polarization-sensitive reflectometry, *i.e.* option for measurement of both *R*_*s*_ and *R*_*p*_ components. This requires a 360° azimuthal rotation of the sample/detector unit. It has been fully designed in-house up to the constructional drawings. The manufacture and assembly took place at a main contractors site. The motor control is carried out under *LABVIEW* software, while for data acquisition and control of beamline and reflectometer the *SPEC* program is used communicating *via* EPICS variables with all motors and detectors.

**Figure 2:**
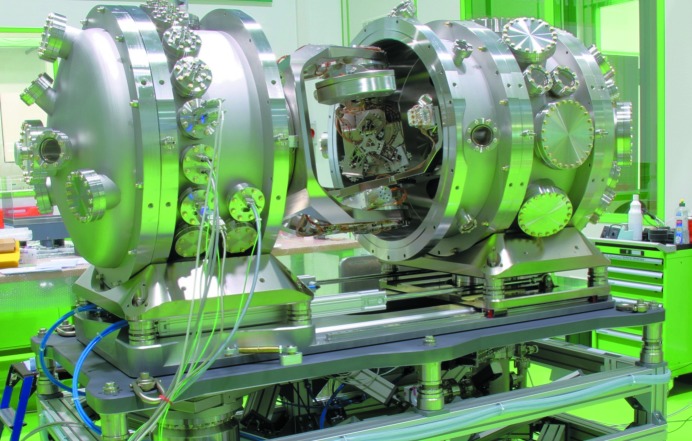
Reflectometer vacuum chamber opened to show the UHV-optical bench. The light comes from the left side.

Fig. 2 ▸ shows the complete reflectometer assembly and its UHV-chamber on the adjustable stand. The individual work packages (vacuum chamber and stand, UHV-optical bench, tripod, load-lock) will be explained in the following subsections.

### 3.1. Vacuum chamber and stand   

The UHV-reflectometer chamber is a 1 m-long stainless steel tube of 0.8 m diameter and two half domes on both sides (volume 2 m^3^). The tube is split into two halves for easy access and maintenance, which are connected by differentially pumped double O-ring sealed flanges. The downstream half of the chamber vessel stands on a rail system connected with a pneumatic drive for easy opening and closing. The base plate glides on a coated Al-plate for easy 2D adjustment and the whole assembly with a mass of 2.1 tons is transported on air cushions. The chamber is pumped by a 2000 l s^−1^ turbomolecular pump reaching a base pressure of <5 × 10^−9^ mbar. To speed up the pump down time to not more than 2–3 h a liquid-nitrogen cold trap and a titanium sublimation pump can additionally be activated.

### 3.2. UHV-optical bench   

The optical specification for the functionality of the reflectometer comprised the following items:

(i) Maximum sample mass 4 kg.

(ii) Maximum sample dimension 360 mm × 60 mm × 60 mm.

(iii) Azimuthal angle scan by 360°.

(iv) Incidence angle scan by 90°.

(v) Detector in-plane scan by 180°.

(vi) Detector off-plane scan by ±5°.

(vii) Full flexibility in sample positioning.

(vii) Option for a variety of detectors.

(ix) Option for sample current measurement (sample electrically insulated).

(x) Load-lock for small samples.

**Figure 3:**
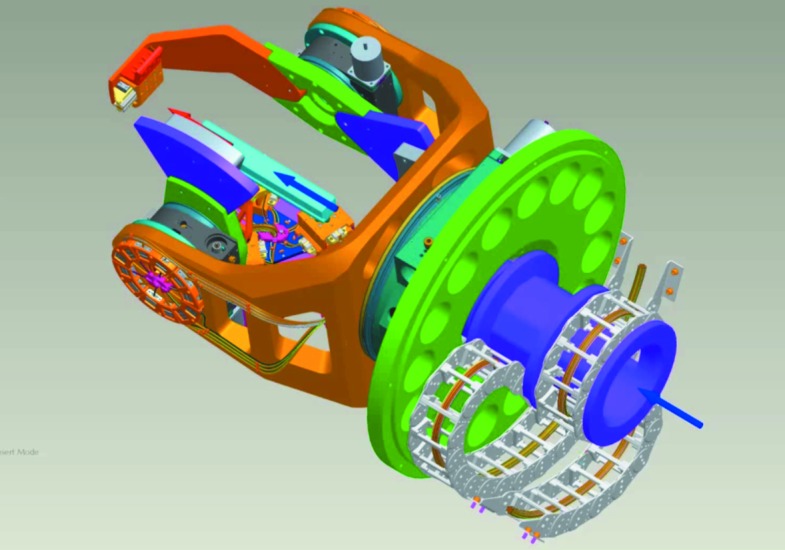
UHV-optical bench showing the three goniometer circles for azimuth, θ and 2θ rotation. The light comes from the right side (blue arrow). The sample is shown in light blue, and the energy chain for the cable guidance in light grey.

This resulted in a UHV-optical bench design which is shown schematically in Fig. 3 ▸. A large stainless steel base plate of 700 mm diameter (green) rigidly attached to the chamber wall holds a large goniometer for the azimuthal rotation φ. This goniometer holds a precisely manufactured and robust U-shaped base plate (total mass 52 kg), onto which two smaller goniometers are mounted opposite to each other and perpendicular to the large goniometer. They are precisely pre-aligned on a common rotation axis for the sample stage θ and the detector arm 2θ. The goniometers realise three circles, and the detector arm holds two translation stages (only one is shown) for out-of-plane movement of a variety of different detectors. This approximates the fourth and fifth circle of the reflectometer. Thus, together with the six axes of the tripod sample alignment stage, we have realised a four-circle 11-axes UHV-diffractometer. All motorized rotations and translations are controlled by rotational or linear encoders and two-stage limit switches. A patent has been granted for a new wiring arrangement of a simple and compact two-stage limit switch requiring only two cables instead of four (Eggenstein & Bischoff, 2012 ▸). All 272 shielded Kapton-insulated wires for the motors, limit switches and encoders of the 11 axes and of the detectors are fed axially through the large goniometer and are guided on a special UHV-energy chain to hinder/prevent twisting during azimuthal rotation by 360°. Table 4 ▸ summarizes the main parameters of the reflectometer circles and axes.

### 3.3. Tripod   

For precise positioning of large and heavy samples a novel tripod system was developed in-house, which is compact and UHV-compatible. This unit allows adjustment of the sample base plate in six degrees of freedom: translations *T*_*x*_, *T*_*y*_, *T*_*z*_ and rotations *R*_*x*_, *R*_*y*_, *R*_*z*_. Three cross slides, each a combination of two perpendicularly arranged saddle slides, are activated by six piezo-ceramic motors with a resolution of 100 nm. Three aluminium legs, which are connected by flexural pivots on one side and by cardan joints on the other side, support the sample base plate. All translational motions are controlled by linear encoders and by two-stage limit switches. The individual scan-ranges for translation and rotation, however, are dependent on each other. A large-area surface scan of the sample reduces the rotational freedom considerably. Table 4 ▸ summarizes the main parameters of the tripod axes.

### 3.4. Load-lock   

The reflectometer will incorporate a magazine store for up to ten samples in a separate small vacuum chamber attached to the main chamber, and a load-lock for rapid sample transfer and change. This will be provided for small and thin samples up to a size of 50 mm × 50 mm × 10 mm only. The sample transfer will be carried out by a motorized linear translation with automatic magnetic fixing and unfixing of the samples which are mounted in standardized frames. This novel technique requiring one linear translation has only recently been patented (Eggenstein, 2013 ▸). Larger samples need to be changed by venting of the vacuum chamber and installing them manually onto the tripod base plate. The pump-down time to the 10^−7^ mbar range is no longer than 3 h.

## 4. Beamline and reflectometer performance   

### 4.1. Photon flux   

**Figure 4:**
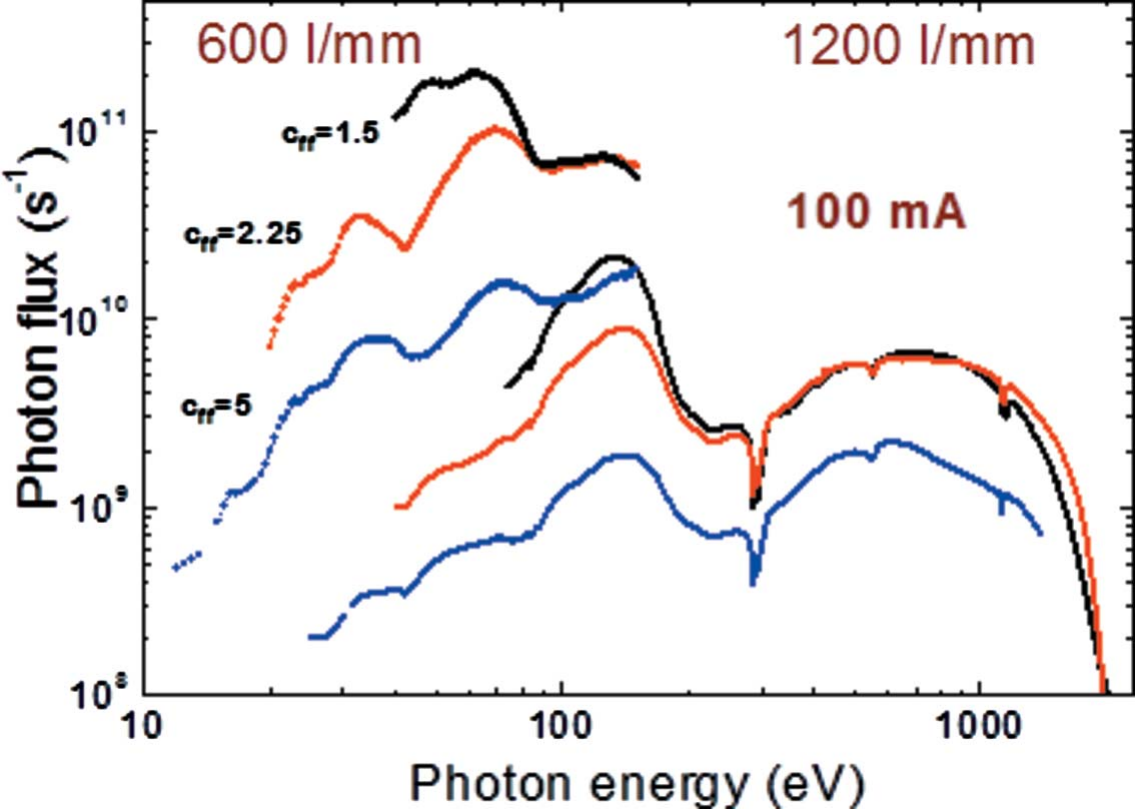
Photon flux of the optics beamline for both gratings and for different *c*_ff_ factors measured with a GaAsP-photodiode detector in the reflectometer. Numbers are given for 300 mA ring current, 100 µm exit slit and 0.5 mrad × 1 mrad (v × h) beamline acceptance.

The available photon flux for both gratings of the SX700 monochromator is presented in Fig. 4 ▸. Shown are the results obtained at different *c*_ff_ values. The figure demonstrates the high-flux (small *c*_ff_) and high-resolution (large *c*_ff_) operation. At standard beamline settings (*c*_ff_ = 2.25, 100 µm exit slit) a photon flux of >10^11^ s^−1^ for energies below 200 eV, >10^10^ s^−1^ in the range 200–1000 eV, and >10^9^ s^−1^ above 1 keV at full acceptance and at a typical ring current of 300 mA top-up operation are demonstrated.

### 4.2. Resolving power   

**Figure 5:**
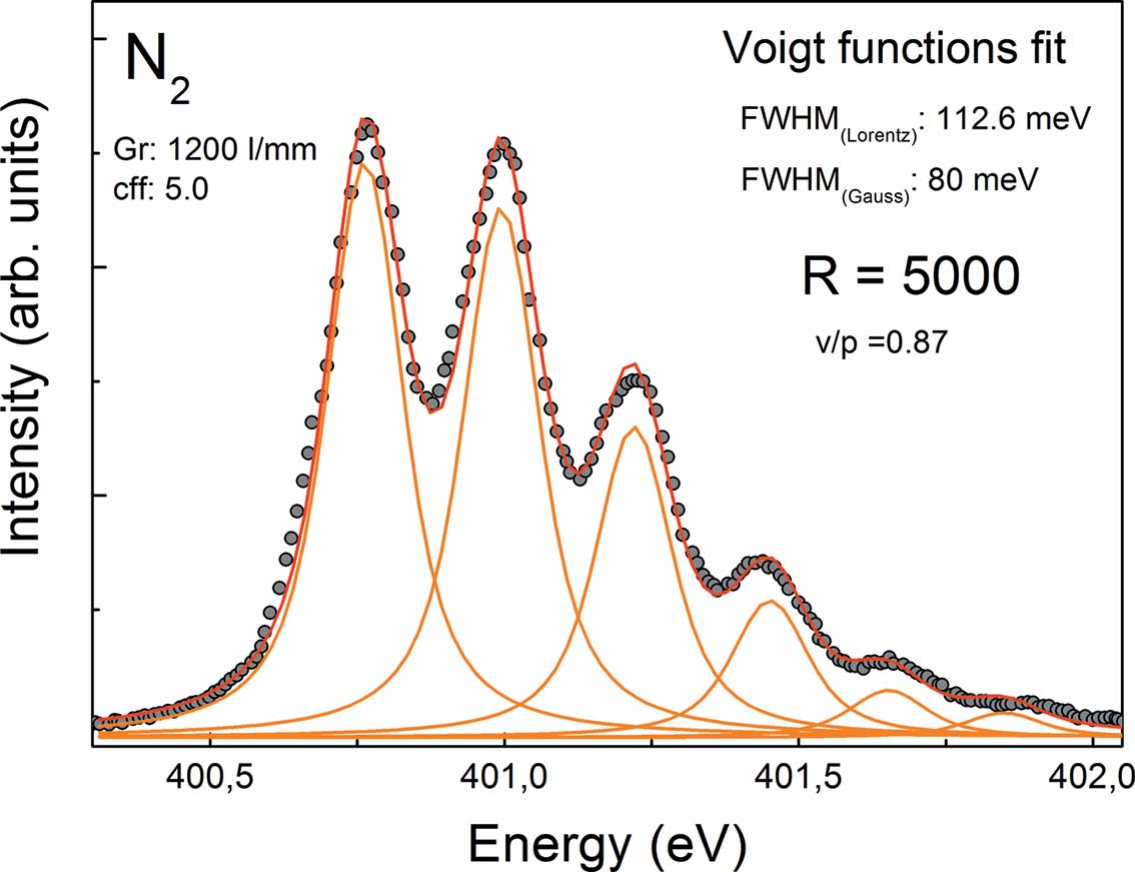
Nitrogen 1*s* absorption lines measured with the ionization chamber of the beamline. The ratio of first valley to third peak is v/p = 0.87. A Voigt function fit delivers a resolving power of *E*/Δ*E* = 5000 at 400 eV.

**Figure 6:**
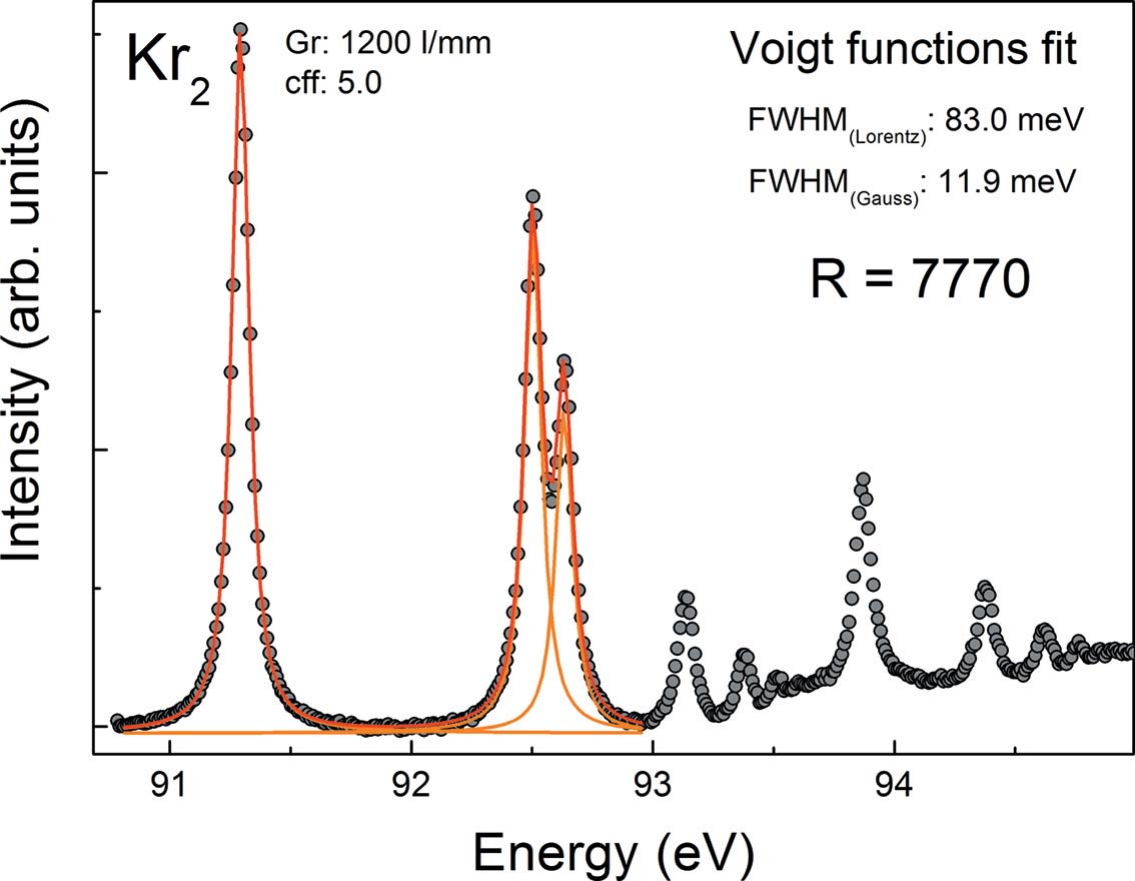
Krypton absorption spectrum in the range of the 3*d*_3/2_ and 3*d*_5/2_ thresholds measured with the ionization chamber of the beamline. A fit delivers a resolving power of *E*/Δ*E* = 7770 around 92 eV.

The energy calibration and resolution tests are routinely performed by absorption spectroscopy on suitable gases (Ar, Kr, Ne, N_2_) with an ionization chamber mounted permanently between the refocusing mirror and FSU chamber. Fig. 5 ▸ shows the N_2_ 1*s* absorption lines which are routinely used to measure the resolving power. At *c*_ff_ = 5, a Voigt function fit to the absorption spectrum yields a resolving power of *E*/Δ*E* = 5000 at 400 eV. Similarly the absorption spectrum of gaseous Kr in the range of the 3*d* thresholds (Fig. 6 ▸) yields a resolving power of 7770 at *c*_ff_ = 5 at 92 eV. Thus a moderate resolution of *E*/Δ*E* = 2.000–10.000 has been demonstrated, which is adequate for at-wavelength metrology and reflectometry experiments for which this beamline has been designed.

### 4.3. Focus size   

**Figure 7:**
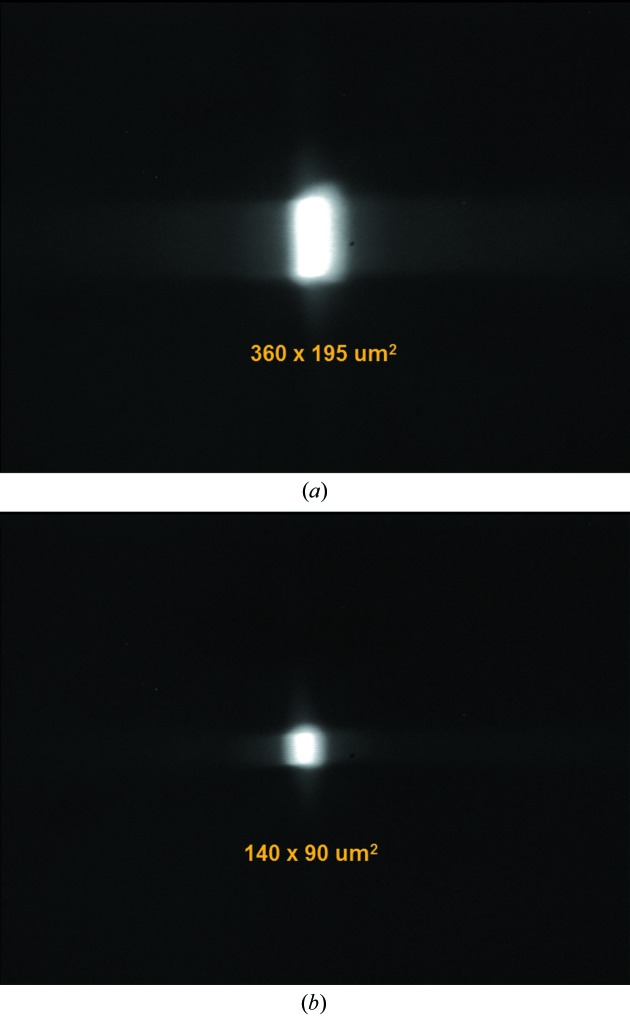
Beamline focus at the position of the sample in the reflectometer chamber (*a*) at standard beamline settings [500 eV, *c*_ff_ = 2.25, exit slit 100 µm, acceptance 0.56 mrad × 0.85 mrad (v × h)] and (*b*) at a reduced beamline acceptance of 0.28 mrad × 0.28 mrad and an exit slit of 30 µm. The focus size is 380 µm × 185 µm and 140 µm × 80 µm, respectively

The beam has an intermediate horizontal focus in front of the exit slit, a vertical focus at the exit slit and is then re­focused. All these focus positions and focus sizes were determined with a focus measuring chamber, which is essentially a fluorescence screen and a CCD camera which can be translated longitudinally along the light beam. After alignment of the M1, M3 and M4 mirrors and of the exit slit position to achieve best energy resolution, Fig. 7(*a*) ▸ shows the focus size of 360 µm × 195 µm FWHM (v × h). The vertical size is proportional to the exit slit which is magnified by the refocusing mirror M4 by a factor of 2.8 to achieve sufficient spacing between the last mirror M4 and the rather large reflectometer chamber. The horizontal focus is determined by the source size which is imaged approximately 1:1. The minimum measured beam size was 140 µm × 90 µm for reduced aperture and exit slit setting (Fig. 7*b* ▸). Due to the low divergence of the beam (<0.5 mrad × 3.6 mrad) the footprint at the detector site 310 mm further downstream of the focus is not significantly increased.

### 4.4. Polarization   

**Figure 8:**
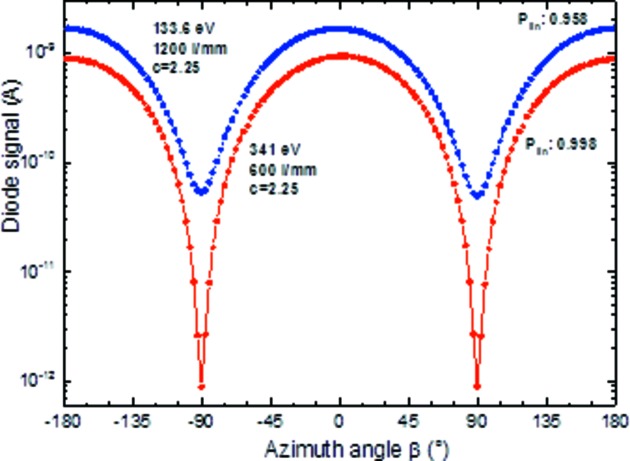
Reflected intensity of two Brewster-angle multilayer mirrors (Cr/Sc and Cr/C) as a function of azimuthal angle to determine the linear polarization of the beam at two energies (134 eV and 341 eV, respectively). The beamline vertical opening angle was set differently (0.6 and 0.15 mrad, respectively) and thus the net linear polarization. Multilayer parameters, blue curve: Cr/C with 40 periods and a period thickness of 6.64 nm operated at a Brewster angle of 45°; red curve: Cr/Sc with 400 periods and a period thickness of 2.57 nm operated at a Brewster angle of 45°

**Figure 9:**
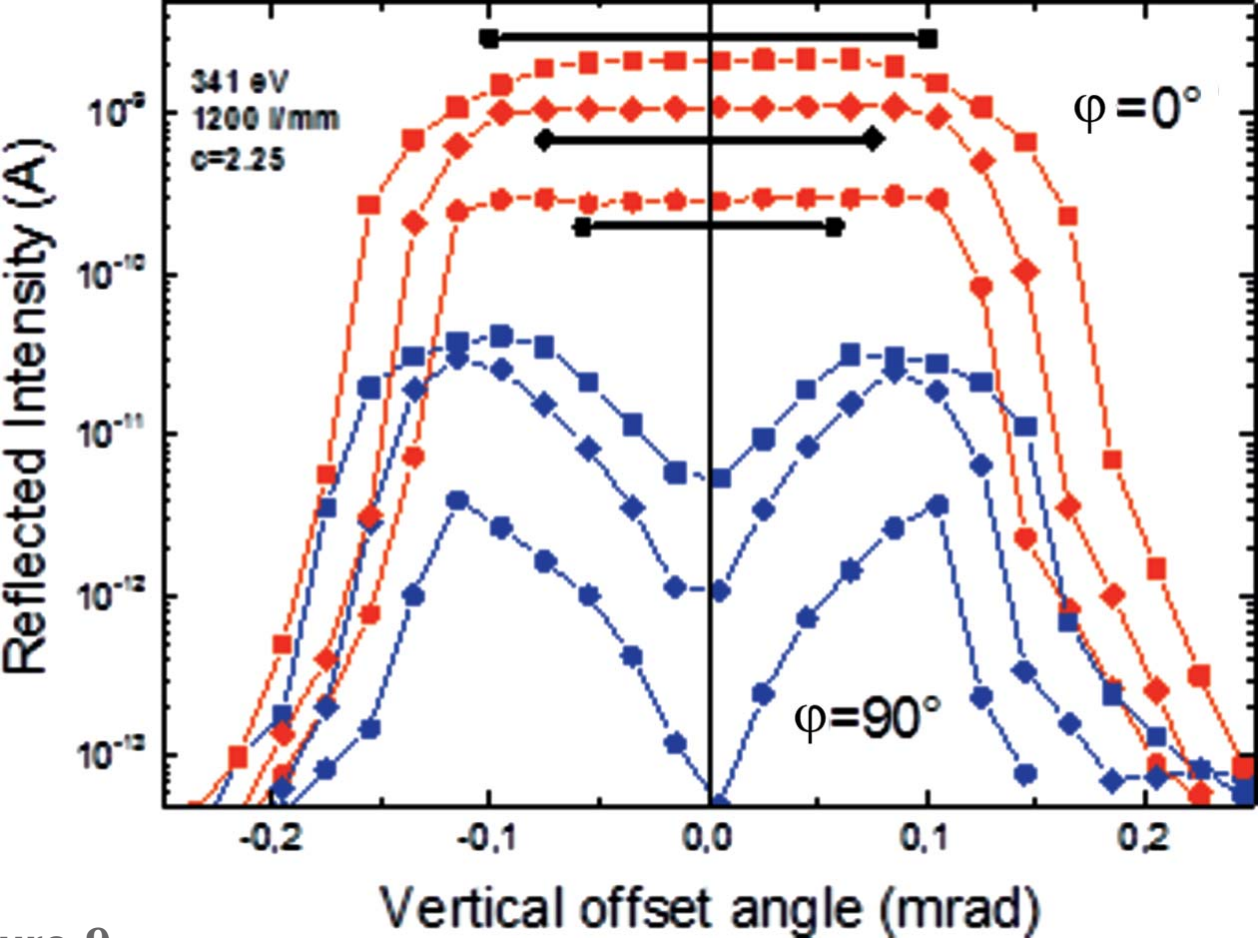
Intensity *I*_par_ and *I*_perp_ in *s*- and *p*-polarization geometry (θ = 0° and 90°, respectively) as a function of the vertical offset angle of the incident beam for different opening angles (black lines). Multilayer parameters: Cr/Sc with 400 periods and a period thickness of 2.57 nm operated at a Brewster angle of 45°. *I*_perp_, which has a phase retardation of ±90° with respect to *I*_par_, is zero in the orbit plane.

**Figure 10:**
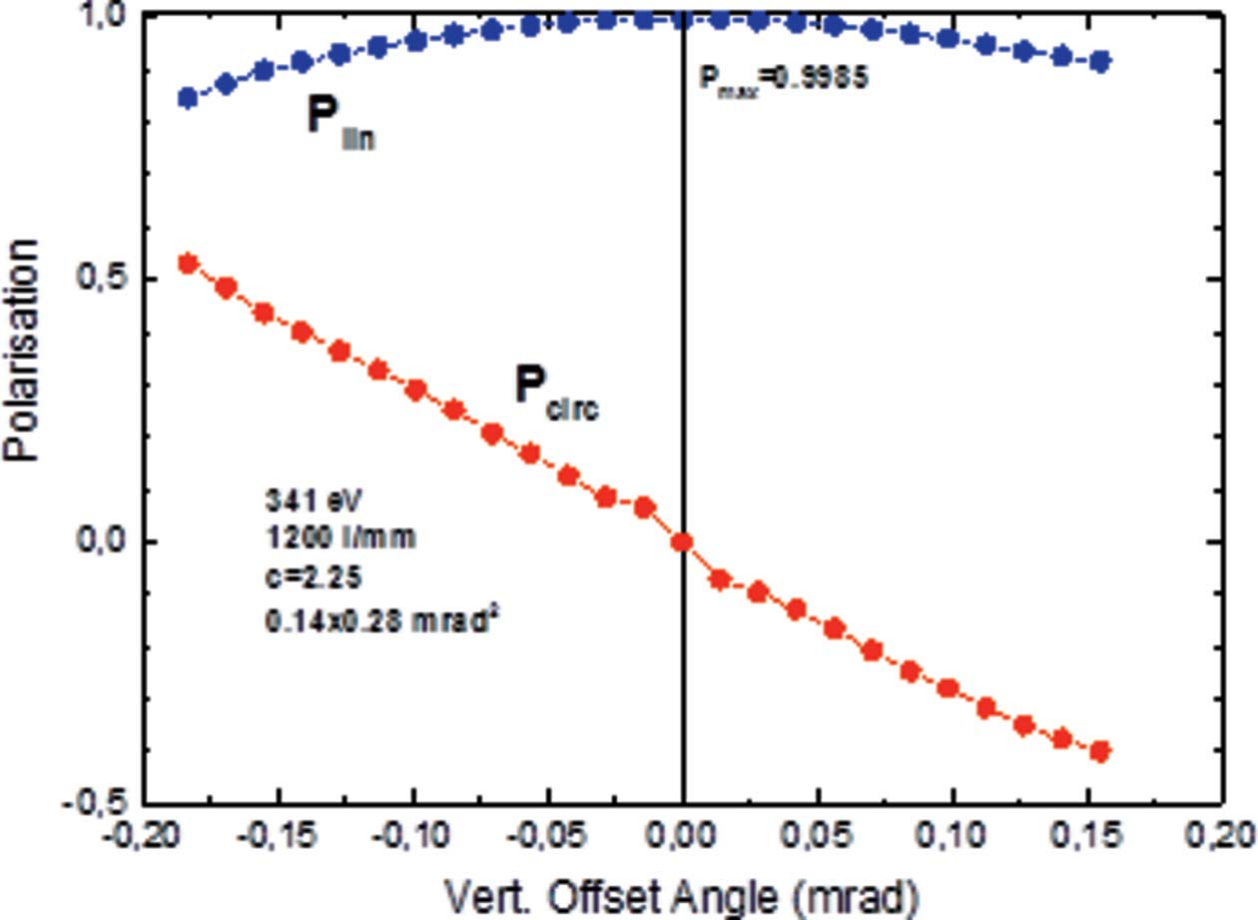
Degree of linear and circular polarization as a function of the vertical offset angle as obtained from the data of Fig. 9 ▸ (circles, 0.14° opening angle). The sign of the circular component has been added artificially (not measured). For multilayer parameters , see Fig. 9 ▸ caption.

The polarization of the incident beam can easily be measured with the reflectometer since the samples can be rotated azimuthally around the light direction (φ-scan). Thus, given suitable polarization-sensitive optics such as mirrors or multilayers operating under the Brewster angle, the linear polarization is determined by polarization scans as shown in Fig. 8 ▸. Here, multilayer mirrors of Cr/Sc and Cr/C, with periods of 400 and 40, respectively, and a *d*-spacing of 2.57 nm and 6.64 nm, respectively, were set to the Brewster angle near θ = 45°, and the intensity reflected at 2θ = 90° was measured with a GaAs-photodiode as a function of the azimuthal angle φ. Obviously the φ and the light beam axis are perfectly aligned to each other over the full angular range of φ since there is no instrumental asymmetry between φ = −180°, 0° and 180°, and similarly for the two Brewster minima at φ = ±90°. The resulting polarization is determined as the difference (*I*_max_ − *I*_min_) divided by the sum (*I*_max_ + *I*_min_), the radiation is completely linearly polarized as expected, *P*_lin_ = 0.998 and 0.958, respectively, which corresponds to a horizontal polarization plane of the dipole radiation. This is a very precise method to determine the orbit plane of the electron beam in the storage ring. The linear polarization is maximum within the electron beam plane and becomes elliptical outside. A vertical aperture scan across the beam orbit in *s*- and in *p*-polarization geometry (φ = 0°, parallel component *I*_par_) and φ = 90° (perpendicular component *I*_perp_), respectively (Fig. 9 ▸), defines the orbit plane as the position of maximum linear polarization (Fig. 10 ▸). The smaller the vertical opening angle the larger the linear polarization. A maximum value of *P*_lin_ = 0.9985 has been determined at a photon energy of 341 eV. No significant change of the beam polarization with energy is expected, but this has not yet been determined experimentally. Assuming no unpolarized background, the circular polarization is determined by *P*_circ_ = 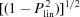. The beamline will be established for optional use of elliptically polarized off-plane radiation soon according to the polarization steering techniques described by Kachel *et al.* (2015 ▸).

### 4.5. Reflectometer alignment   

The internal alignment of the UHV-optical bench with 11 motorized axes has been extensively characterized with precision optical tooling techniques: theodolite (Leica TMA 5100), electronic autocollimator (Elcomat 3000), laser tracker (Leica AT901-B) and a 90°-corner mirror as double-reflector on top of the tripod stage. The results for the tripod positioning accuracy in the arcsec range are given by Eggenstein *et al.* (2014 ▸). The rotation axes for sample angle θ, detector angle 2θ and azimuthal angle φ were characterized with respect to angular mismatch and offset. Table 5 ▸ gives the results for the mutual tilt and displacement of the respective axes. The axes-tilt stays below 0.025° (90 arcsec) and the axes displacement is less than 60 µm. Thus a pointing stability during a sample scan of less than 60 µm and 0.025° is achieved.

**Figure 11:**
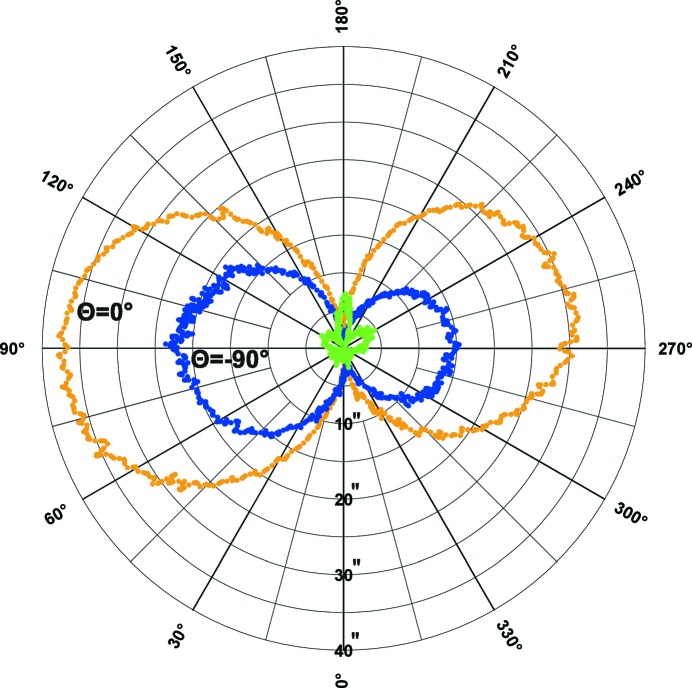
Autocollimation of the φ-goniometer stage taken with a 90°-corner-mirror on the tripod stage, taken at θ = 0° (orange curve) and 90° (blue curve), respectively. The green curve is obtained by a correction function applied to the tripod (feedback).

By tracing the autocollimation direction of a mirror at the sample position on top of the tripod as a function of the φ-goniometer position (azimuthal scan) the data in Fig. 11 ▸ are obtained. The orange and blue curves were determined with the tripod rotating vertically (θ = 0°) and horizontally (θ = 90°), respectively. Thus the stiffness of the tripod under different gravitational situations [standing (φ = 0°), hanging (φ = 180°), horizontally (φ = ±90°)] can be determined by this method. The maximum measured wobble is 0.01° (35 arcsec) which is partly due to the intrinsic stiffness of the Huber goniometer (15") and is well within the specification. However, having measured these data the wobble can be compensated for by an adequate readjustment of the tripod position using a look-up table. This is demonstrated by the green curve where the tripod position has been repositioned during scanning to a minimum deviation of less than 10 arcsec.

## 5. Metrology experiments   

### 5.1. Multilayer mirrors   

**Figure 12:**
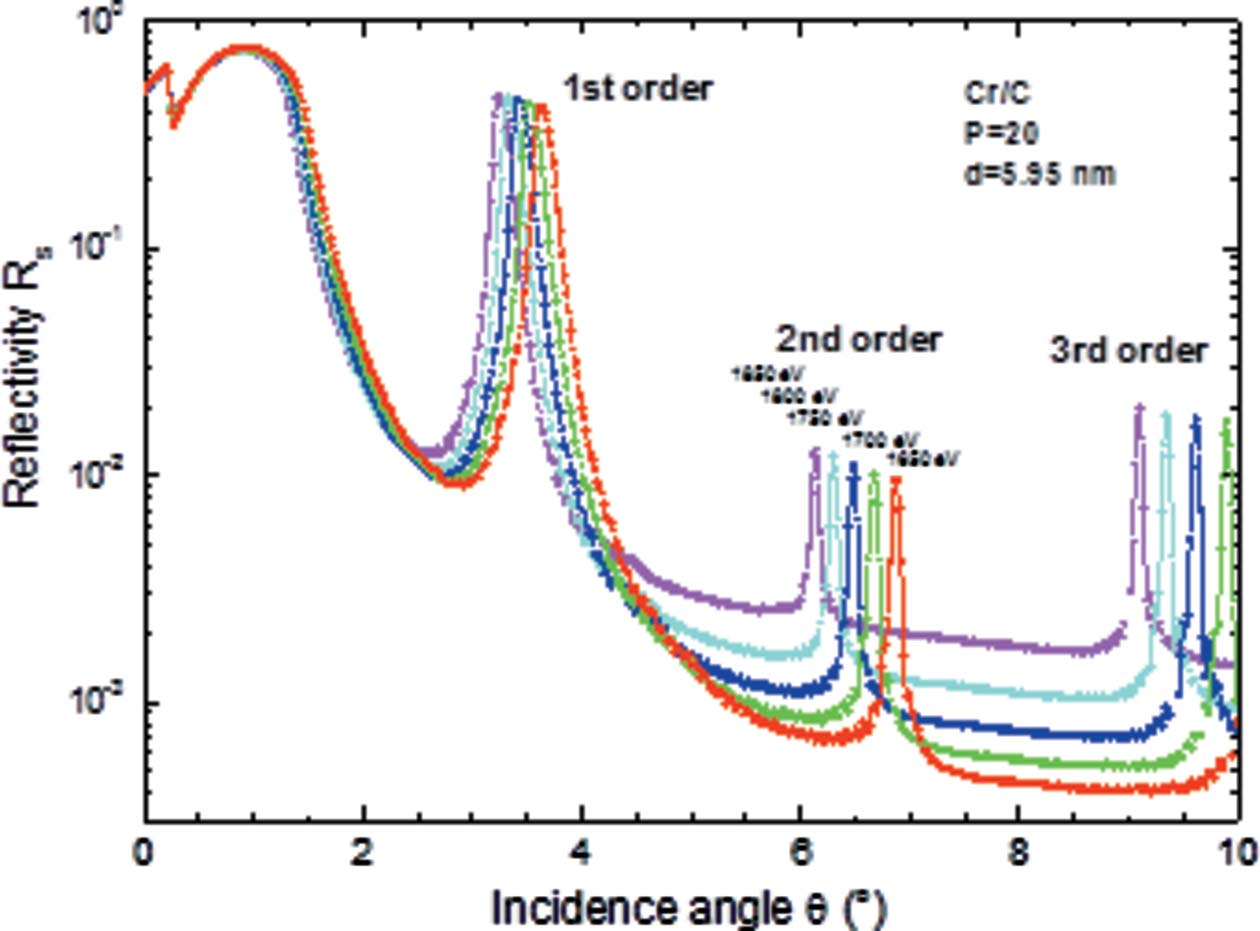
Cr/C-multilayer reflectivity curves (θ-2θ scans) for different photon energies in the XUV-range in first, second and third diffraction order. All curves start at *R* = 0.5 at θ = 0° which is a hint for a perfect alignment with the tripod stage. Multilayer parameters: Cr/C with 20 periods and a period thickness of 5.95 nm.

The reliability of reflectivity data depends very critically on the spectral purity of the incident beam. This was tested by round-robin multilayer mirrors which were previously measured at the PTB-metrology laboratory and with our polarimeter chamber. The overall agreement for a Mo/Si multilayer was better than 0.5%. Fig. 12 ▸ shows a reflectivity scan of a Cr/C multilayer at selected energies in the XUV range to demonstrate the measurement accuracy and the perfect alignment of the sample with respect to the incident beam made possible by the tripod sample adjustment stage.

### 5.2. Diffraction gratings   

**Figure 13:**
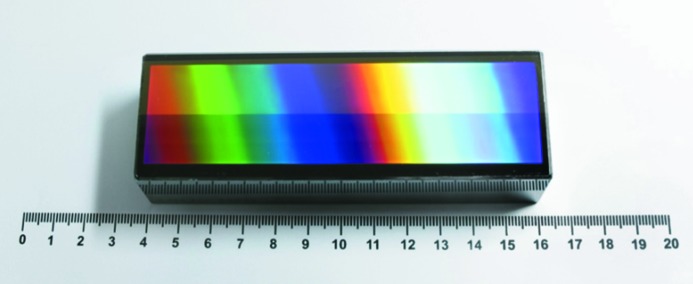
A blazed diffraction grating of 600 lines mm^−1^ recently produced at HZB for the BESSY-II optics beamline.

**Figure 14:**
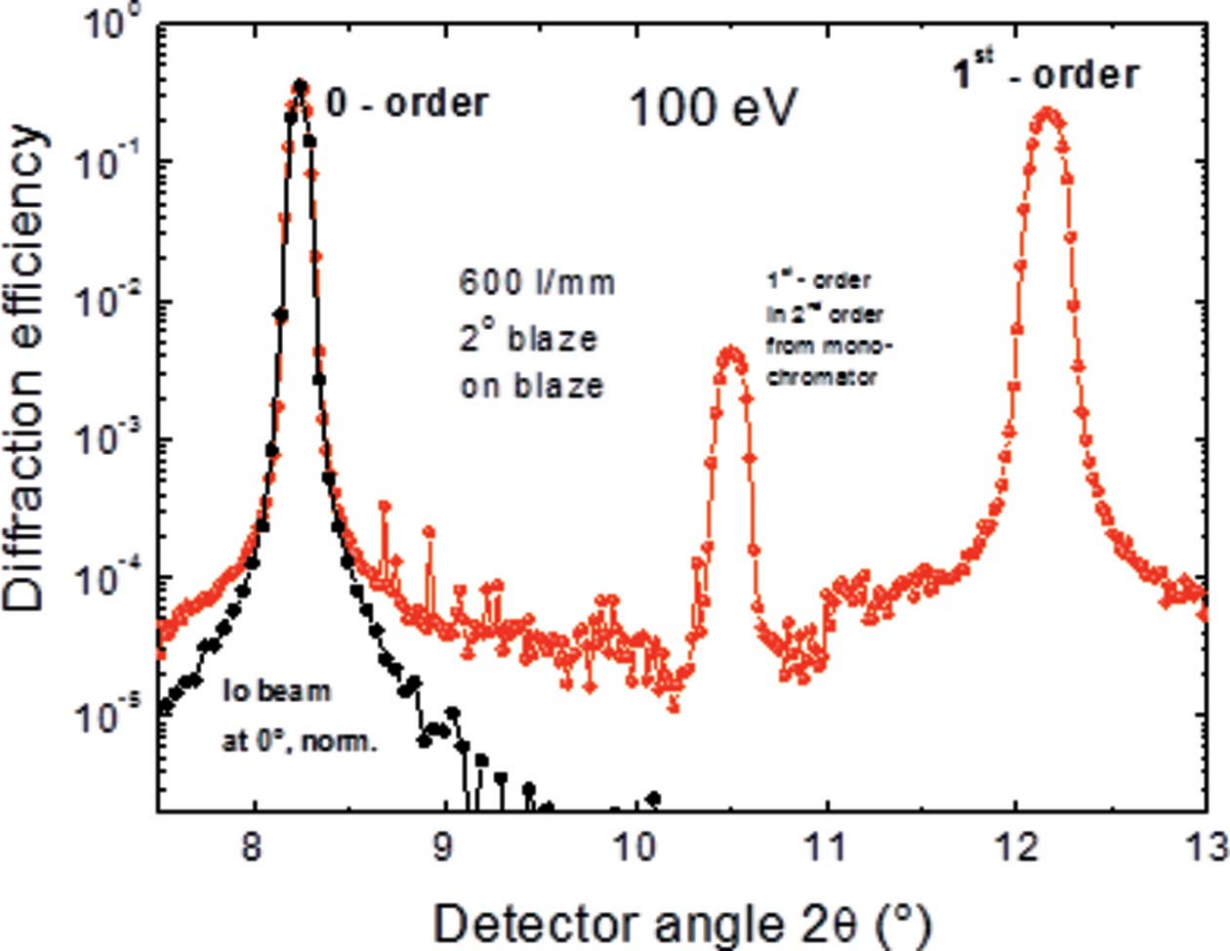
Diffraction efficiency of a blazed diffraction grating of 600 lines mm^−1^ in zero and first order at 100 eV, operated on-blaze. The Io-beam profile (shifted and normalized to the zero-order peak) is also shown for comparison (black points). Second-order radiation in the incident beam (photon energy 200 eV) which is not suppressed creates a first-order diffraction peak at 2θ = 10.5°.

**Figure 15:**
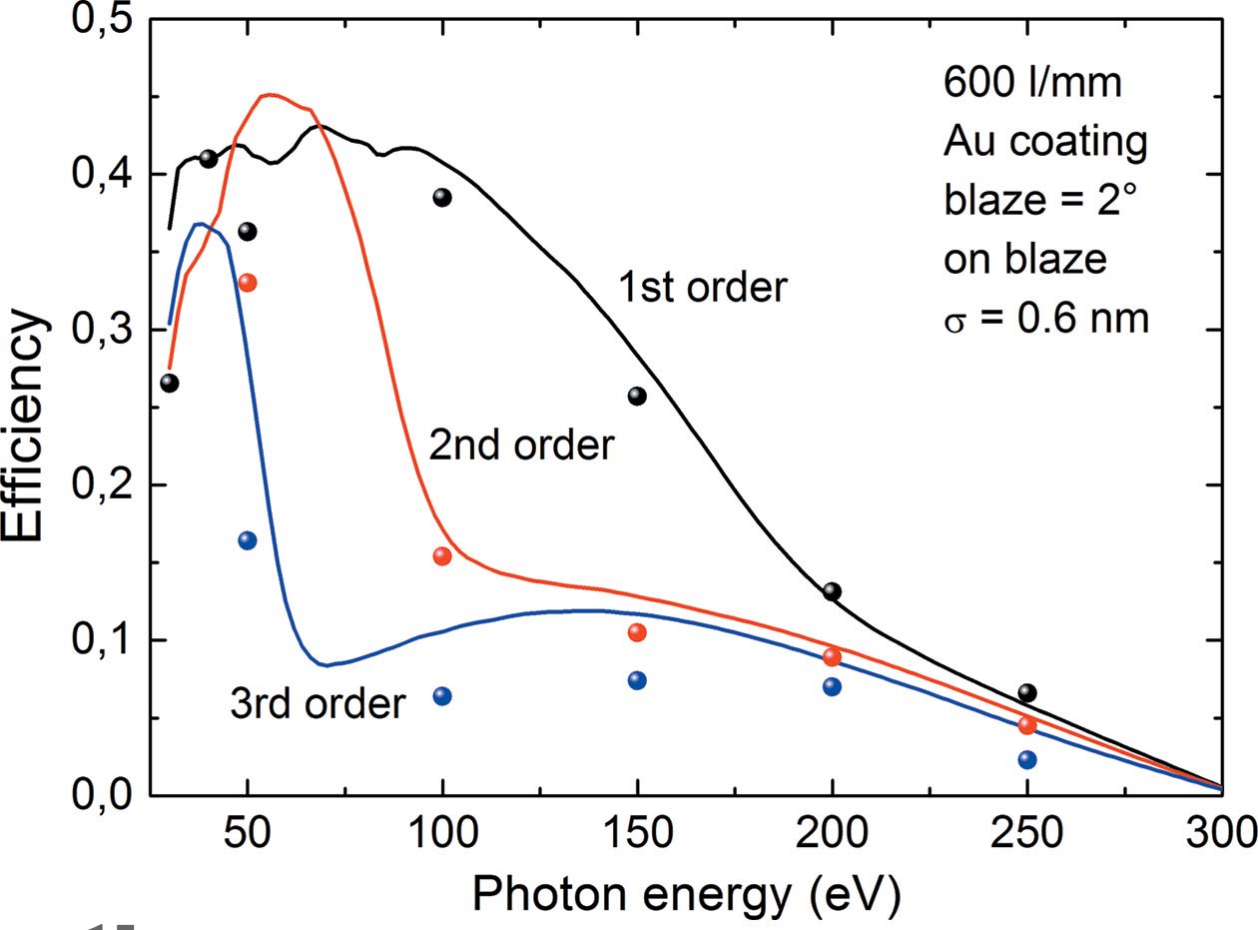
Integrated efficiencies of the 600 lines mm^−1^ blazed grating for the first, second and third order in comparison with calculation using *REFLEC* (Schäfers & Krumrey, 1996 ▸).

The main purpose of this at-wavelength metrology facility is the final control and quality check of the in-house-produced diffraction gratings. One of the first ever produced gratings at HZB was made to replace the 30-year-old ZEISS gratings in the SX700 monochromator which was revitalized for the optics beamline described here. Fig. 13 ▸ shows a photograph of this grating and Fig. 14 ▸ shows a typical 2θ-reflectivity curve at a fixed incidence angle at fixed photon energy of 100 eV for the 600 lines mm^−1^ grating which has a blaze angle of 2°. The figure shows the zero and first diffraction order. First-order diffraction of second-order incident radiation is also seen. The signal-to-noise ratio due to both grating and beamline stray light is better than four orders of magnitude. Fig. 15 ▸ shows the full data set of efficiency measurements obtained by evaluation of such 2θ-scans. The efficiency was determined by integrating the peak area of the respective diffraction order since the peak width differs with the chosen *c*_ff_ factor. Integrated efficiency values of up to 40% were recorded in good agreement with calculations using the *REFLEC* program (Schäfers & Krumrey, 1996 ▸).

### 5.3. Multilayer gratings   

**Figure 16:**
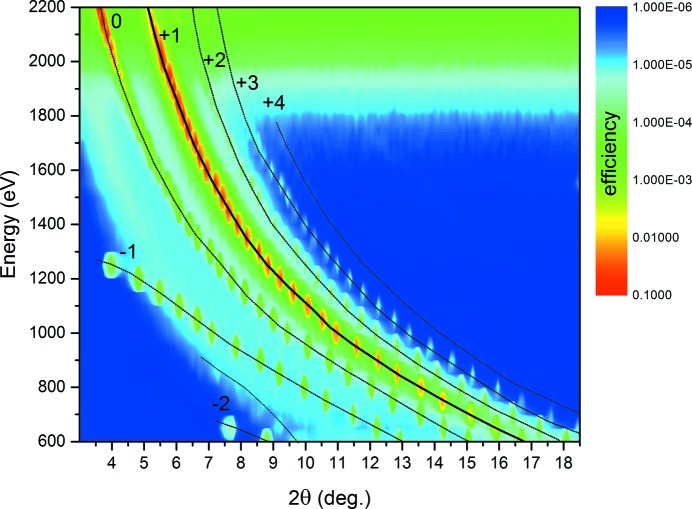
Two-dimensional map of the diffracted intensity (energy *versus* detector angle 2θ) of a Cr/C multilayer-coated blazed grating (2000 lines mm^−1^) recently produced at HZB for the soft X-ray range. All diffraction orders from −1 to 4 are visible (Senf *et al.*, 2016 ▸).

Though the idea of combining a highly reflecting multilayer with a diffraction grating to achieve highest efficiencies is a very old one (Jark, 1986 ▸), this idea was revitalized recently by various research groups (Voronov *et al.*, 2010 ▸; Choueikani *et al.*, 2014 ▸). Combining the multilayer Bragg equation with the grating equation for blazed surfaces one finds a solution for the connection of multilayer period and blaze angle at which the on-blaze grating efficiency should be increased over a large energy range. Thus, such a grating should be suitable to be incorporated into modern synchrotron radiation beamlines. Such a multilayer-coated blazed grating has been realised recently in-house and the efficiency was measured at-wavelength (Fig. 16 ▸). The grating and multilayer parameters were chosen such that the energy range covers the soft X-rays: the difficult region between 1 and 5 keV, between grating and crystal monochromator operation. Very high efficiencies of 35% at 2 keV and 55% at 4 keV were measured. Detailed results will be published elsewhere (Senf *et al.*, 2016 ▸).

### 5.4. Zone plates   

**Figure 17:**
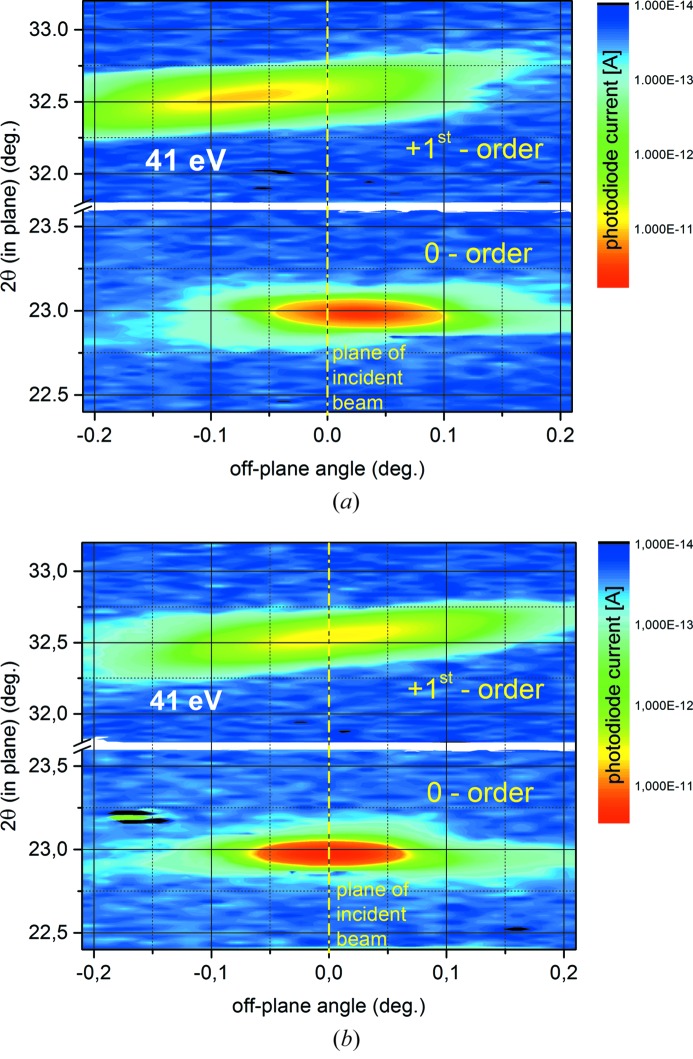
Two-dimensional maps of the reflected intensity (in-plane *versus* off-plane detector angle) of a reflection zone plate recently produced at HZB for the UV-range before (*a*) and after (*b*) yaw-alignment.

Reflective zone plates (RZPs) are very attractive XUV dispersive and focusing optical elements for monochromators or XUV spectrometers, which have a high transmission and preserve the time structure of the radiation in the femto­second range. Such RZP elements are developed at our institute (Erko *et al.*, 2010 ▸) and the final characterization of their efficiency is performed with reflectometry. Similarly to a VLS grating the RZP has a variable periodic structure and a strong curvature of the lines. Therefore a precise alignment to the incident beam is mandatory. This is possible by using the six-axes alignment tool of the tripod, which is demonstrated in Fig. 17 ▸. Here zero- and first-order RZP diffraction is shown for a non-aligned (Fig. 17*a* ▸) and aligned RZP (Fig. 17*b* ▸). By a normal rotation and a change of the sample’s roll, the dispersion plane is rotated into the slit-plane of the detector.

## 6. Conclusions   

We have described a new UV- and XUV-optics beamline at the BESSY-II synchrotron radiation facility which is coupled permanently to a novel versatile 11-axes UHV-reflectometer situated in a moderate clean-room hutch. This new facility is optimized and dedicated to at-wavelength metrology, which we have shown to be a powerful, non-destructive and indispensable infrastructure tool for development, characterization and final control of UV/XUV optics such as nano-optical or nano-electronic devices, multilayer structures or zone plates. At-wavelength performance data cannot be obtained by any other method. In particular, this equipment is used for the development of new optical elements and especially for the in-house-manufactured diffraction gratings of which the fabrication is well established now. The novel five-circle reflectometer allows high-precision metrology on large-scale samples on a short-term access at 24 h/7 d semi-automated operation. The beamline delivers completely linearly polarized light, but it will be upgraded soon to the use of off-plane bending-magnet radiation with a high degree of left- and right-hand circular polarization. This will enable ellipsometry and polarimetry applications on polarization-sensitive and helical substances as well. The beamline is also open for external user operation on other subjects such as (non)-resonant reflection or photoemission spectroscopy for investigations of internal material structure or (buried) interface quality.

## Figures and Tables

**Table 1:** BESSY-II source characteristics of the dipole section DIP 1.1

Electron energy (GeV)	1.7
Magnetic field (T)	1.3
Bending radius (m)	4.35
Power on first optical element (300 mA) (W)	20
Critical energy (keV)	2.5
Source horizontal size (σ_*x*_) (mm)	0.096
Source vertical size (σ_*y*_) (mm)	0.047
Source horizontal divergence ( 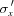 ) (µrad)	300
Source vertical divergence ( 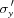 ) (µrad)	20

**Table 2:** Parameters of the focusing mirrors of the beamline

Optical element	M1	M3	M4
Shape	Toroidal	Cylindrical	Toroidal
Surface size (L × W) (mm)	1000 × 60	1000 × 60	350 × 30
Substrate	Si	Si	Si
Coating (thickness) (nm)	Au (40)	Au (40)	Au (40)
Tangential radius *R* (mm)	339578	194000	82725
Sagittal radius ρ (mm)	1040.3	470.3	52.8
Grazing incidence angle (°)	2°	2°	2°
Source distance (h / v) (mm)	15000	– / ∞	2975 / 1036
Focus distance (h / v) (mm)	9795 / ∞	– / 6734	2800
Roughness σ (nm r.m.s.)	0.25	0.23	0.25
Slope error (tangential / sagittal) (µrad r.m.s.)	0.85 / 3.25	0.9 / 3.5	1.5 / 5.0

**Table 3:** Parameters of the SX700 monochromator optics

Optical element	PG1	PG2	M2
Shape	Plane grating	Plane grating	Plane mirror
Optical surface size (L × W) (mm)	120 × 40	120 × 40	600 × 40
Substrate	Si	Si	Zerodur
Coating (thickness) (nm)	Au (40)	Au (40)	Au (40)
Tangential radius (km)	95	94	>300
Line density (lines mm^−1^)	1200	600	–
Blaze angle (°)	1.1°	2°	–
Grazing incidence angle (°)	1–24°	1–24°	1.5–13°
Roughness σ (nm r.m.s.)	0.3	0.6	0.5
Slope error (µrad r.m.s.)	0.2	0.3	0.3

**Table 4:** Parameters of the 11 reflectometer axes

Axis	Hardware	Range	Positioning accuracy
Sample azimuth angle φ	Huber 430	−180° to 180°	3.6"
Sample incidence angle θ	Huber 411	−90° to 90°	3.6"
Detector angle 2θ	Huber 411	−180° to 180°	3.6"
Detector off-plane (two axes)	Ceramic motors	−25 mm to 25 mm (−4° - 4°)	50 nm
Sample adjustment *T*_*x*_, *T*_*y*_, *T*_*z*_	Ceramic motors	−20 mm to 20 mm (not simulated)	500 nm
Sample adjustment *R*_*x*_, *R*_*y*_, *R*_*z*_	Ceramic motors	−10° to 10° (not simulated)	1"

**Table 5:** Alignment of goniometer axes

Axis 1	Axis 2	Tilt (°)	Distance of closest approach (mm)
θ	2θ	179.979	0.014
θ	β	90.025	0.049
2θ	β	89.994	0.057
